# Dr. Greenhill’s Prospectus

**Published:** 1845-10

**Authors:** W. A. Greenhill

**Affiliations:** Oxford


A friend and correspondent has handed us the following "Prospectus," with a request that we would give it a place in the Examiner. Dr. Greenhill is a learned and greatly respected member of the medical profession, and there can be no doubt, so far as it may be in his power to render it so, that the proposed publication will be able, interesting, and impartial.

PROSPECTUS.

1. It is proposed (with God's assistance) to publish in a series the lives of those Physicians who have been most eminent for their piety, in whatever age and country they may have lived.

2. The profits (if any) arising from these publications will be given eventually to some Medical Charity.

3. In this undertaking the Editor will be happy to receive literary assistance from such of his friends as may take an interest in the work.

4. As the whole of the present expense and risk falls upon the Editor alone, he will gladly accept any donations or subscriptions in aid of the design from persons who may feel an interest in it.

5. The lives will of course vary much in length, some forming each a volume of itself, and others constituting a distinct class consisting of much shorter notices. The volumes will not be published in any particular order, but they will be strictly uniform in size, type, &c.

6. The number of volumes to be published annually must depend on the amount of money received either from the sale of the works, or from the donations of friends.

7. The Editor will be answerable for the general accuracy and fidelity of the narratives, for the tone and spirit of the whole work, and for the selection of the lives to be published; and accordingly he will be deeply pained if any man of piety and good sense shall consider either that he has inserted in his list any name but those of Physicians really fearing God and loving Christ, or that he has published any life written in a low or unchristian spirit; but, as it is proposed to comprehend Physicians of all ages and countries, and not merely those who have been members of the Church of England, (still less those who have belonged to this or that particular party in it,) it is manifest that neither the Editor nor the Writers are to be considered responsible for the particular theological opinions which any of these individuals may have held.

W. A. Greenhill, M. D.

Oxford, *All Saint's Day*, 1844.

